# *FADS* genetic and metabolomic analyses identify the ∆5 desaturase (FADS1) step as a critical control point in the formation of biologically important lipids

**DOI:** 10.1038/s41598-020-71948-1

**Published:** 2020-09-28

**Authors:** Lindsay M. Reynolds, Rahul Dutta, Michael C. Seeds, Kirsten N. Lake, Brian Hallmark, Rasika A. Mathias, Timothy D. Howard, Floyd H. Chilton

**Affiliations:** 1 Division of Public Health Sciences, Department of Epidemiology and Prevention, Wake Forest School of Medicine, Winston-Salem, NC 27157 USA; 2 Department of Urology, Wake Forest School of Medicine, Winston-Salem, NC 27157 USA; 3 Department of Internal Medicine/Molecular Medicine, and the Wake Forest Institute of Regenerative Medicine, Wake Forest School of Medicine, Winston-Salem, NC 27157 USA; 4 Department of Nutritional Sciences, University of Arizona, Tucson, AZ 85719 USA; 5 The BIO5 Institute, University of Arizona, Tucson, AZ 85719 USA; 6 Division of Allergy and Clinical Immunology, Department of Medicine, Johns Hopkins University, Baltimore, MD 21224 USA; 7 Department of Biochemistry, Wake Forest School of Medicine, Winston-Salem, NC 27157 USA

**Keywords:** Lipids, Metabolomics, Biomarkers, Diseases, Molecular medicine, Genetic association study, Heritable quantitative trait

## Abstract

Humans have undergone intense evolutionary selection to optimize their capacity to generate necessary quantities of long chain (LC-) polyunsaturated fatty acid (PUFA)-containing lipids. To better understand the impact of genetic variation within a locus of three *FADS* genes (*FADS1*, *FADS2*, and *FADS3*) on a diverse family of lipids, we examined the associations of 247 lipid metabolites (including four major classes of LC-PUFA-containing molecules and signaling molecules) with common and low-frequency genetic variants located within the *FADS* locus. Genetic variation in the *FADS* locus was strongly associated (*p* < 1.2 × 10^–8^) with 52 LC-PUFA-containing lipids and signaling molecules, including free fatty acids, phospholipids, lyso-phospholipids, and an endocannabinoid. Notably, the majority (80%) of *FADS*-associated lipids were not significantly associated with genetic variants outside of this *FADS* locus. These findings highlight the central role genetic variation at the *FADS* locus plays in regulating levels of physiologically critical LC-PUFA-containing lipids that participate in innate immunity, energy homeostasis, and brain development/function.

## Introduction

**Figure 1:**
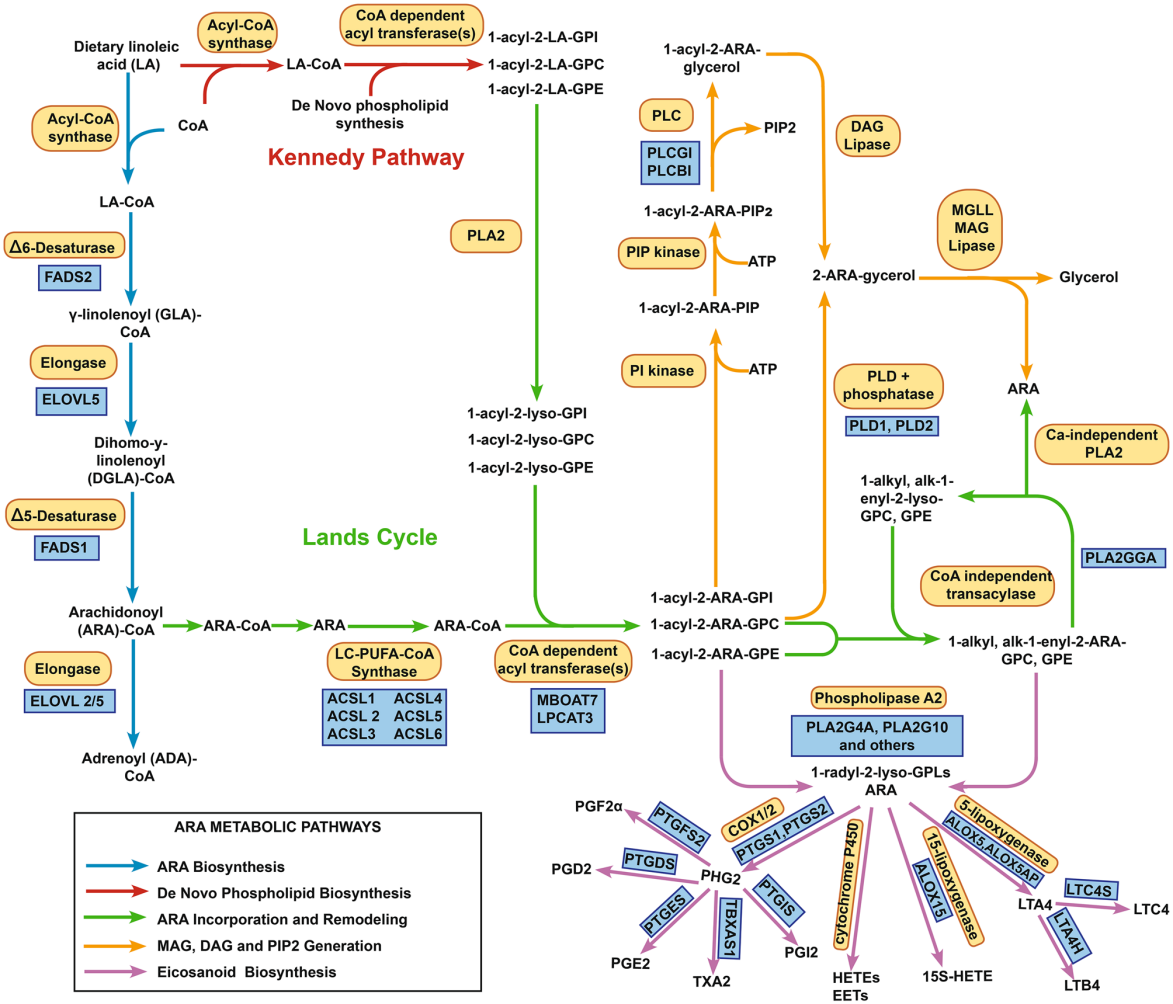
Biosynthesis and Metabolism of n-6 PUFAs and LC-PUFAs. The arrows in the metabolic pathway is depicted in four colors: blue—biochemical steps responsible for the conversion of LA-CoA to ARA-CoA and ADA-CoA; red—biochemical steps in the Kennedy pathway; green—biochemical steps in the Lands cycle and ARA remodeling into 1-alkyl and 1-alk-1-enyl linked phospholipids; orange—phospholipase C- and phospholipase D- and diglyceride lipase-induced steps leading to PIP2 and 2-ARA-glycerol; purple—phospholipase A2-induced steps leading to eicosanoid biosynthesis.

Long chain (20–22 carbon; LC-) polyunsaturated fatty acids (PUFAs) linked to complex lipids (such as phospholipids [PL], lyso-phospholipids [lyso-PL], glycerides, cholesterol esters, and endocannabinoids) or as unesterified (free) fatty acids have structural and functional roles in human physiology and pathophysiology and mediate critical steps in innate immunity, energy homeostasis, brain development, and neurocognitive function^1–4^. Humans cannot synthesize PUFAs, thus PUFAs must be obtained from the diet^5,6^. The majority of dietary PUFAs are plant-based eighteen carbon (18C-) PUFAs, such as linoleic (LA; 18:2, n-6) and α-linolenic (ALA; 18:3, n-3) acids. Once absorbed, 18C-PUFAs are taken up by tissues such as the liver and rapidly converted to 18C-PUFA-CoenzymeAs (CoA) by acyl-CoA synthetase, which then have the capacity to be converted (by desaturase and elongase activities) into biologically-active n-6 and n-3 LC-PUFAs^7,8^. The capacity of tissues to synthesize LC-PUFAs is associated with genetic variation within the fatty acid desaturase (*FADS*) locus on chromosome 11 (11q12.2–q13.1), containing the genes *FADS1*, *FADS2* and *FADS3*. *FADS1* and *FADS2* encode the rate-limiting ∆5 and ∆6 desaturase enzymes, respectively, in the LC-PUFA biosynthesis pathway (Fig. 1)^9–17^. *FADS3* encodes a desaturase whose biological role is less clear. There are dramatic differences in frequencies of genetic variation within the *FADS* locus of different human populations that are not under neutral drift, but bear strong signatures of selection^18–22^. This is likely due to the critical importance of LC-PUFAs as structural complex lipids and as signaling molecules in many physiological systems, and thus the necessity for humans to regulate LC-PUFA levels as they adapted to very diverse diets.

After LC-PUFAs like arachidonic acid (ARA) are synthesized, they are taken from circulation and primarily incorporated into phospholipids of cells and tissues utilizing a highly integrated set of biochemical pathways (Fig. 1). Landmark studies by Kennedy and Weiss in 1956^23,24^ demonstrated that 18C-PUFAs are incorporated into phospholipids during de novo synthesis, shown at the top of Fig. 1. However, this pathway didn’t account for how n-6 or n-3 LC-PUFAs such as ARA, eicosapentaenoic acid (EPA; 20:5, n-3) and docosahexaenoic acid (DHA; 22:6, n-3) became highly enriched at the sn-2 position of cellular phospholipids. Lands and colleagues demonstrated that newly-formed phospholipids are rapidly remodeled by removing 18C-PUFAs and replacing them with these LC-PUFAs^25,26^. It was then discovered that LC-PUFAs could be moved from 1-ester-linked to 1-ether-linked (1-alkyl and 1 alk-1-enyl) phospholipids utilizing a CoA-independent transacylase^27–29^. Collectively, these biochemical steps result in well over 100 individual PUFA- and LC-PUFA-containing phospholipid and lyso-phospholipid molecular species, depending upon the cells or tissues being examined. Once formed, LC-PUFA-containing phospholipid molecular species have biophysical properties, signaling roles, and also serve as substrates for a variety of phospholipases, including numerous isoforms of phospholipases A_2_, C, and D. Subsequent oxygenase enzymes generate diverse classes of biologically-active lipids including prostaglandins, thromboxanes, hydroxyeicosatetraenoic acids, epoxyeicosatrienoic acids, leukotrienes, lipoxins, resolvins, protectins, maresins, and endocannabinoids^30–35^.

Although numerous studies have looked for associations between LC-PUFA levels and genetic variation or clinical traits^36^, it is typically not the LC-PUFAs themselves that impact membrane structure and signaling properties but the LC-PUFA-containing complex lipids or metabolites, such as PLs and lyso-PLs, and the generation of signaling molecules, such as unesterified LC-PUFAs, LC-PUFA metabolites, and endocannabinoids (e.g., 2-arachidonoyl glycerol). Notably, the FADS1 catalyzed desaturation of dihomo-gamma-linolenic acid (DGLA, 20:3) forms ARA necessary for the biosynthesis of ARA-containing complex lipids shown in Fig. 1. Similar or identical biochemical steps are utilized for the formation and metabolism of complex lipids containing n-3-LC-PUFAs^7,37,38^.

Most genetic and evolutionary studies to date have largely focused on total 18C-PUFA and LC-PUFA levels found across all molecular entities, without attention to the complex lipids from which they were derived. Typically, in these studies, fatty acids are removed from complex lipids utilizing a saponification step that occurs before lipid analyses^39^. While these previous studies have provided important information on associations between *FADS* locus variations with total PUFAs and LC-PUFAs, these methods do not have the capacity to determine which of the 50–100 18C-PUFA and LC-PUFA-containing circulating complex lipids are most impacted by variation in the *FADS* gene locus or evaluate the relative importance of the *FADS* locus versus numerous genes that code for other enzymes in these complex pathways (Fig. 1). To better understand the relationship between *FADS* locus variation and levels of complex lipids, we examined the associations between individual 18C-PUFA and LC-PUFA lipid molecular species and genetic variants using data from a recent whole-genome sequencing study of genetic influences on the blood metabolome^40^.

## Results

### Impact of FADS variation on levels of PUFA- and LC-PUFA-containing complex lipids, unesterified fatty acids, and signal molecules

**Figure 2:**
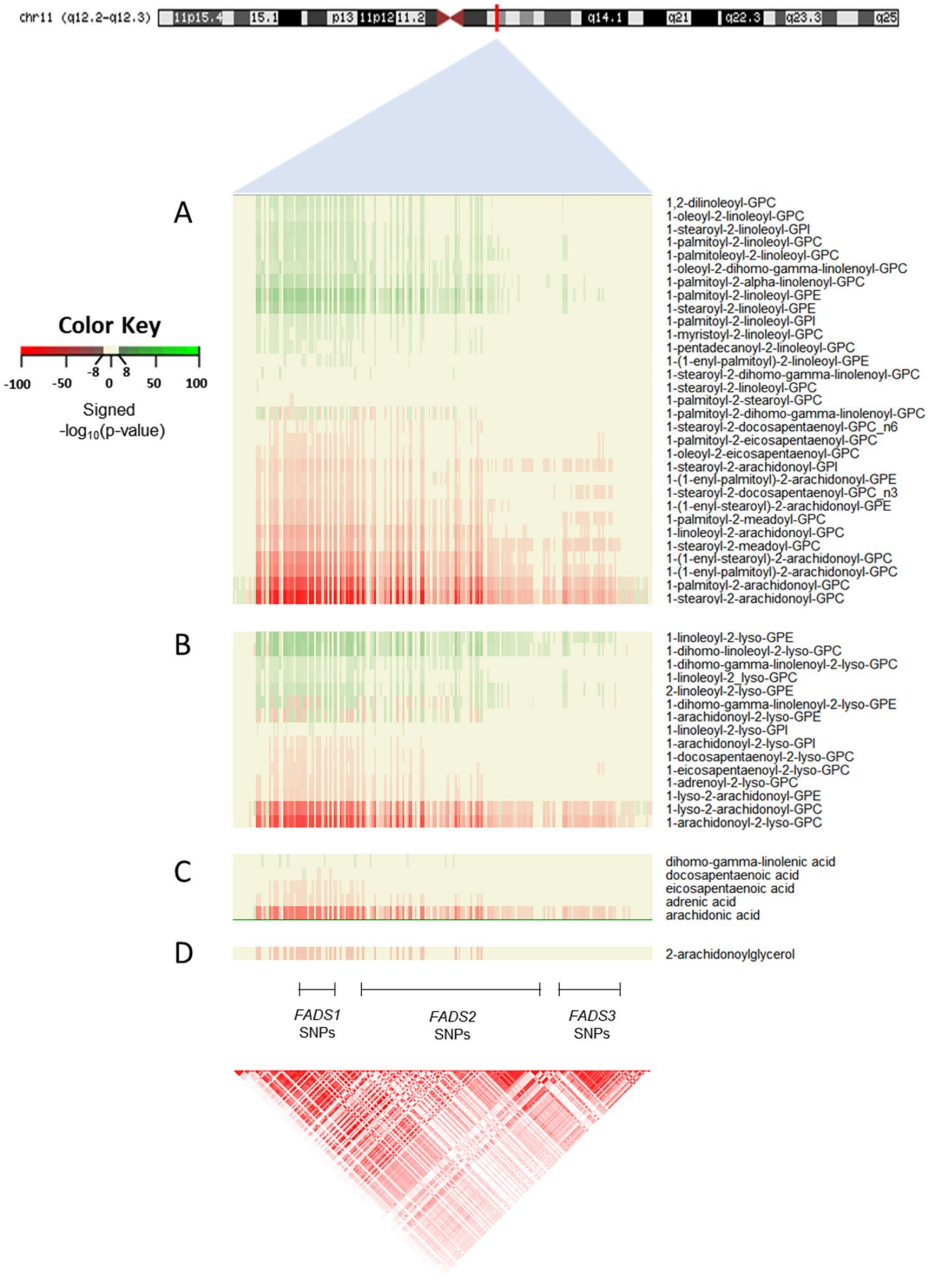
*FADS* locus genetic variation associations with lipid metabolites. Heatmaps depicting the significance (− log_10_[*p* value]) and effect direction for associations between 52 metabolites and chromosome 11 SNPs in the *FADS* locus (± 100 kb) that reached a significance threshold (*p* < 1.2 × 10^–8^). The effect direction and significance of association between the alternative (alt) allele and the metabolite levels are depicted by color (see color key). Green color indicates significantly higher metabolite levels associated with increasing numbers of the SNP alt allele. In contrast, red depicts lower metabolite levels associated with increasing numbers of the alt allele. The neutral beige color indicates associations *p* ≥ 1.2 × 10^–8^. The heatmaps are separated by four major lipid classes including from top to bottom: (**A**) PL (31 metabolites), (**B**) lyso-PL (15 metabolites), (**C**) free fatty acid (5 metabolites), and (**D**) endocannabinoid (1 metabolite). The LD plot represents R^2^ values using the CEU population from the 1,000 Genomes Project.

Whole-genome sequencing and metabolomic profiling was previously conducted in 1,960 adult participants of the TwinsUK study by Long et al.^40^ to examine the relationships between human genetic variation and blood levels of 644 metabolites, providing an important resource for further exploration. To better understand the influence of genetic variation surrounding *FADS1* and *FADS2* on circulating levels of several classes of lipids, we utilized results from the Long et al. study and investigated highly significant association results between single nucleotide polymorphisms (SNPs) in the *FADS* locus (Chr 11: 61,692,980–61,992,051, build hg38, including a ± 100 kb flanking region) and lipid metabolite levels. As depicted in Fig. 2, 52 lipids were associated with *FADS* genetic variation (208 SNPs, *p* < 1.2 × 10^–8^, minor allele frequency [MAF] ranging from 0.024 to 0.484), including n-6, n-3, and n-9 PUFA- and LC-PUFA-containing lipid molecular species, representing four major lipid classes: phospholipids, lyso-phospholipids, free fatty acids, and the endocannabinoid 2-arachidonyl glycerol (2-AG). Figure 2 reveals a key pattern among lipid metabolites based on the direction of the associations. Molecular species containing PUFAs and LC-PUFAs that are metabolically upstream of the FADS1 ∆-5 desaturase step are green (e.g., LA and dihomo-gamma-linolenic acid [DGLA; 20:3, n-6]), indicating significantly higher metabolite levels with each copy of the minor allele. In contrast, lipids containing highly unsaturated n-6 and n-3 LC-PUFAs (e.g., ARA) that are downstream of the FADS1 step are red, illustrating that metabolite levels are significantly lower with each copy of the minor allele across all four lipid classes. Notably, there were some unexpected bi-directional effects observed between *FADS* locus SNPs and some of the metabolites, including 1-palmitoyl-2-dihomo-gamma-linolenoyl-GPC (upstream, before FADS1), 1-dihomo-gamma-linolenoyl-2-lyso-GPE (upstream, before FADS1), 1-arachidonoyl-2-lyso-GPE (downstream, after FADS1), and docosapentaenoic acid (downstream, after FADS1).

### Associations of PUFA- and LC-PUFA-containing complex lipids, unesterified fatty acids and signaling molecules with rs174564

The peak association identified between lipid levels and *FADS* locus variants was with rs174564 (chr 11: 61,820,833, build hg38), which resides in a long linkage disequilibrium (LD) block within the *FADS* locus. Specifically, the peak association was an inverse association between the number of copies of rs174564 minor allele (G) and 1-stearoyl-2-ARA-sn-glycero-3-phosphocholine, which had an effect size of − 0.71 (*p* = 3.52 × 10^–91^). Differences in gene expression of *FADS1* and *FADS2,* are associated with the rs174564, where the minor allele (G) is associated with lower *FADS1* and higher *FADS2* expression in multiple tissues by the Genotype-Tissue Expression (GTEx) project^43^, a publicly available platform to examine the relationships between genetic variation and gene expression across a wide range of human tissues. The peak association SNP rs174564 was chosen as a representative variant for further examination, as the high LD in this region precludes identification of specific functional SNPs through genetic effects alone.

Tables 1 and 2 list the 48 PUFA and LC-PUFA-containing lipid molecular species linked to the peak-association SNP, rs174564. Overall, the rs174564 minor allele (G) is associated with higher levels of 19 phospholipid and lyso-phospholipid molecular species that contain either LA or DGLA, which fall upstream of the FADS1 ∆5 enzymatic step in the LC-PUFA biosynthetic pathway, with effect sizes ranging from 0.21 to 0.89 (Table 1). The one exception, 1-ARA-2-lyso-GPE contains ARA and is on the downstream side of the FADS1 step. In contrast, Table 2 shows molecular species from four major classes of lipids (PL, lyso-PL, free fatty acids, and an endocannabinoid) that have lower levels associated with each copy of the rs174564 minor allele (G). All n-6 or n-3 LC-PUFA lipid molecular species linked to phospholipids, lyso-phospholipids, free fatty acids, and an endocannabinoid found in Table 2 are located downstream of FADS1. LC-PUFA-containing lipids that are most strongly inversely associated (i.e., effect sizes ≤ − 0.40) with the rs174564 minor allele include the molecular species 1-palmitoyl-2-ARA-GPC, *p* = 8.05 × 10^–72^; 1-(1-enyl-palmitoyl)-2-ARA-GPC, *p* = 3.78 × 10^–48^; 1-(1-enyl-stearoyl)-2-ARA-GPC, 3.90 × 10^–43^; 1-stearoyl-2-meadoyl-GPC, *p* = 8.30 × 10^–28^; 1-linoleoyl-2-ARA-GPC, *p* = 4.10 × 10^–29^; lyso-phosphatidylcholine (1-ARA-2-lyso-GPC, *p* = 2.72 × 10^–73^; and 1-lyso-2-ARA-GPC, *p* = 8.21 × 10^–56^) as well an unesterified arachidonic acid (*p* = 3.55 × 10^–51^). Several plasmalogen PLs such as 1-enyl-palmitoyl and 1-enyl-stearoyl are also inversely associated with the minor allele (G) at rs174564. These PLs contain a fatty alcohol with a vinyl-ether bond at the *sn*-1 position and have been shown to be highly enriched in LC-PUFAs at the *sn*-2 position of the glycerol backbone in inflammatory and neoplastic cells^41,42^. Importantly, there was also an inverse association between several *FADS* locus SNPs including rs174564 and the endocannabinoid, 2-ARA glycerol.

**Table 1:** Direct associations between number of copies of rs174564 minor allele (G) and serum lipids, *p* < 1.2 × 10^–8^.

Type	Biochemical	GWAS *p* value	Effect size	n
n-6 PUFA-containing lyso-PL	1-Linoleoyl-2-lyso-GPC (18:2)	9.93 × 10^–13^	0.26	1958
n-6 PUFA-containing lyso-PL	1-Lyso-2-linoleoyl-GPE (18:2)	1.33 × 10^–17^	0.27	1940
n-6 PUFA-containing lyso-PL	1-Linoleoyl-2-lyso-GPE (18:2)	6.16 × 10^–32^	0.40	1959
n-6 LC-PUFA-containing lyso-PL	1-Dihomo-linolenoyl-2-lyso-GPE (20:3)	1.79 × 10^–18^	0.89	1951
n-6 LC-PUFA-containing lyso-PL	1-Arachidonoyl-2-lyso-GPE (20:4n6)	1.90 × 10^–19^	0.59	1955
n-6 PUFA-containing PL	1-Palmitoyl-2-linoleoyl-GPE (16:0/18:2)	1.00 × 10^–34^	0.41	1959
n-6 LC-PUFA-containing PL	1-Dihomo-linoleoyl-GPC (20:2)	9.90 × 10^–32^	0.42	1957
n-6 PUFA-containing PL	1-Stearoyl-2-linoleoyl-GPE (18:0/18:2)	1.80 × 10^–29^	0.37	1959
n-6 PUFA-containing PL	1-Palmitoyl-2-alpha-linolenoyl-GPC (16:0/18:3n3)	2.28 × 10^–22^	0.33	1959
n-6 PUFA-containing PL	1-Palmitoyl-2-linoleoyl-GPC (16:0/18:2)	2.18 × 10^–17^	0.33	1959
n-6 PUFA-containing PL	1-Stearoyl-2-linoleoyl-GPI (18:0/18:2)	8.63 × 10^–17^	0.28	1955
n-6 PUFA-containing PL	1-Palmitoleoyl-2-linoleoyl-GPC (16:1/18:2)	1.55 × 10^–16^	0.34	1959
n-6 PUFA-containing PL	1,2-Dilinoleoyl-GPC (18:2/18:2)	1.90 × 10^–16^	0.31	1959
n-6 PUFA-containing PL	1-Oleoyl-2-linoleoyl-GPC (18:1/18:2)	1.07 × 10^–13^	0.26	1958
n-6 LC-PUFA-containing PL	1-Oleoyl-2-dihomo-linolenoyl-GPC (18:1/20:3)	1.70 × 10^–12^	0.25	1956
n-6 PUFA-containing PL	1-Palmitoyl-2-linoleoyl-GPI (16:0/18:2)	1.14 × 10^–10^	0.23	1953
n-6 LC-PUFA-containing PL	1-Dihomo-linolenoyl-GPC (20:3n3 or 6)	1.90 × 10^–10^	0.22	1958
n-6 PUFA-containing PL	1-Myristoyl-2-linoleoyl-GPC (14:0/18:2)	6.07 × 10^–10^	0.22	1959
n-6 PUFA-containing PL	1-Pentadecanoyl-2-linoleoyl-GPC (15:0/18:2)	6.65 × 10^–10^	0.21	1959
n-6 PUFA-containing PL	1-(1-Enyl-palmitoyl)-2-linoleoyl-GPE (P-16:0/18:2)	4.26 × 10^–09^	0.22	1957

All of these PUFA or LC-PUFA-containing lipid molecules are upstream of the FADS1 enzyme step except for 1-arachidonoyl-2-lyso-GPE.

**Table 2:** Inverse associations between number of copies of rs174564 minor allele (G) and serum lipids.

Type	Biochemical	GWAS *p* value	Effect size	n
n-6 Endocannabinoid	2-Arachidonoylglycerol (20:4)	2.20 × 10^–10^	− 0.25	1943
n-6 LC-PUFA	Arachidonate (ARA; 20:4n6)	3.55 × 10^–51^	− 0.53	1958
n-6 LC-PUFA	Adrenate (ADA, 22:4n6)	5.59 × 10^–11^	− 0.18	1958
n-3 LC-PUFA	Eicosapentaenoate (EPA; 20:5n3)	6.06 × 10^–09^	− 0.18	1959
n-6 LC-PUFA-containing lyso-PL	1-Arachidonoyl-2-lyso-GPC (20:4n6)	2.72 × 10^–73^	− 0.64	1956
n-6 LC-PUFA-containing lyso-PL	1-Lyso-2-arachidonoyl-GPC (20:4)	8.21 × 10^–56^	− 0.57	1953
n-6 LC-PUFA-containing lyso-PL	1-Lyso-2-arachidonoyl-GPE (20:4)	4.85 × 10^–14^	− 0.32	1924
n-6 LC-PUFA-containing lyso-PL	1-Adrenoyl-2-lyso-GPC (22:4)	6.35 × 10^–13^	− 0.28	1956
n-6 LC-PUFA-containing lyso-PL	1-Arachidonoyl-2-lyso-GPI (20:4)	1.68 × 10^–09^	− 0.22	1958
n-3 LC-PUFA-containing lyso-PL	1-Eicosapentaenoyl-2-lyso-GPC (20:5)	2.66 × 10^–10^	− 0.22	1957
n-3 LC-PUFA-containing lyso-PL	1-Docosapentaenoyl-2-lyso-GPC (22:5n3)	2.93 × 10^–10^	− 0.22	1958
n-6 LC-PUFA-containing PL	1-Stearoyl-2-arachidonoyl-GPC (18:0/20:4)	3.52 × 10^–91^	− 0.71	1957
n-6 LC-PUFA-containing PL	1-Palmitoyl-2-arachidonoyl-GPC (16:0/20:4)	8.05 × 10^–72^	− 0.64	1959
n-6 or n-3 LC-PUFA-containing PL	Phosphatidylcholine (16:0/22:5n3, 18:1/20:4)	1.15 × 10^–46^	− 0.54	
n-6 LC-PUFA-containing PL	1-(1-Enyl-palmitoyl)-2-arachidonoyl-GPC (P-16:0/20:4)	3.78 × 10^–48^	− 0.50	1911
n-6 LC-PUFA-containing PL	1-(1-Enyl-stearoyl)-2-arachidonoyl-GPC (P-18:0/20:4)	3.90 × 10^–43^	− 0.47	1953
n-9 LC-PUFA-containing PL	1-Stearoyl-2-meadoyl-GPC (18:0/20:3n9)	8.30 × 10^–28^	− 0.41	1958
n-6 LC-PUFA-containing PL	1-Linoleoyl-2-arachidonoyl-GPC (18:2/20:4n6)	4.10 × 10^–29^	− 0.40	1955
n-6 LC-PUFA-containing PL	1-Palmitoyl-2-dihomo-linolenoyl-GPC (16:0/20:3n6)	1.79 × 10^–10^	− 0.38	1957
n-9 LC-PUFA-containing PL	1-Palmitoyl-2-meadoyl-GPC (16:0/20:3n9)	1.14 × 10^–20^	− 0.37	1946
n-6 or n-3 LC-PUFA-containing PL	Phosphatidylcholine (18:0/20:5n3, 16:0/22:5n6)	8.58 × 10^–21^	− 0.33	
n-6 LC-PUFA-containing PL	1-(1-Enyl-stearoyl)-2-arachidonoyl-GPE (P-18:0/20:4)	2.91 × 10^–19^	− 0.32	1959
n-6 LC-PUFA-containing PL	1-Stearoyl-2-arachidonoyl-GPI (18:0/20:4)	4.19 × 10^–17^	− 0.32	1956
n-6 LC-PUFA-containing PL	1-(1-Enyl-palmitoyl)-2-arachidonoyl-GPE (P-16:0/20:4)	7.73 × 10^–15^	− 0.29	1958
n-3 LC-PUFA-containing PL	1-Stearoyl-2-docosapentaenoyl-GPC (18:0/22:5n3)	1.02 × 10^–14^	− 0.29	1956
n-6 LC-PUFA-containing PL	1-Stearoyl-2-docosapentaenoyl-GPC (18:0/22:5n6)	4.26 × 10^–10^	− 0.24	1958
n-3 LC-PUFA-containing PL	1-Oleoyl-2-eicosapentaenoyl-GPC (18:1/20:5)	8.51 × 10^–11^	− 0.23	1938
n-3 LC-PUFA-containing PL	1-Palmitoyl-2-eicosapentaenoyl-GPC (16:0/20:5)	3.51 × 10^–10^	− 0.22	1959

These lipid species are all downstream from the FADS1 enzyme step.

### Impact of genome-wide variation on levels of PUFA- and LC-PUFA-containing complex lipids, unesterified fatty acids and signal metabolites

**Figure 3:**
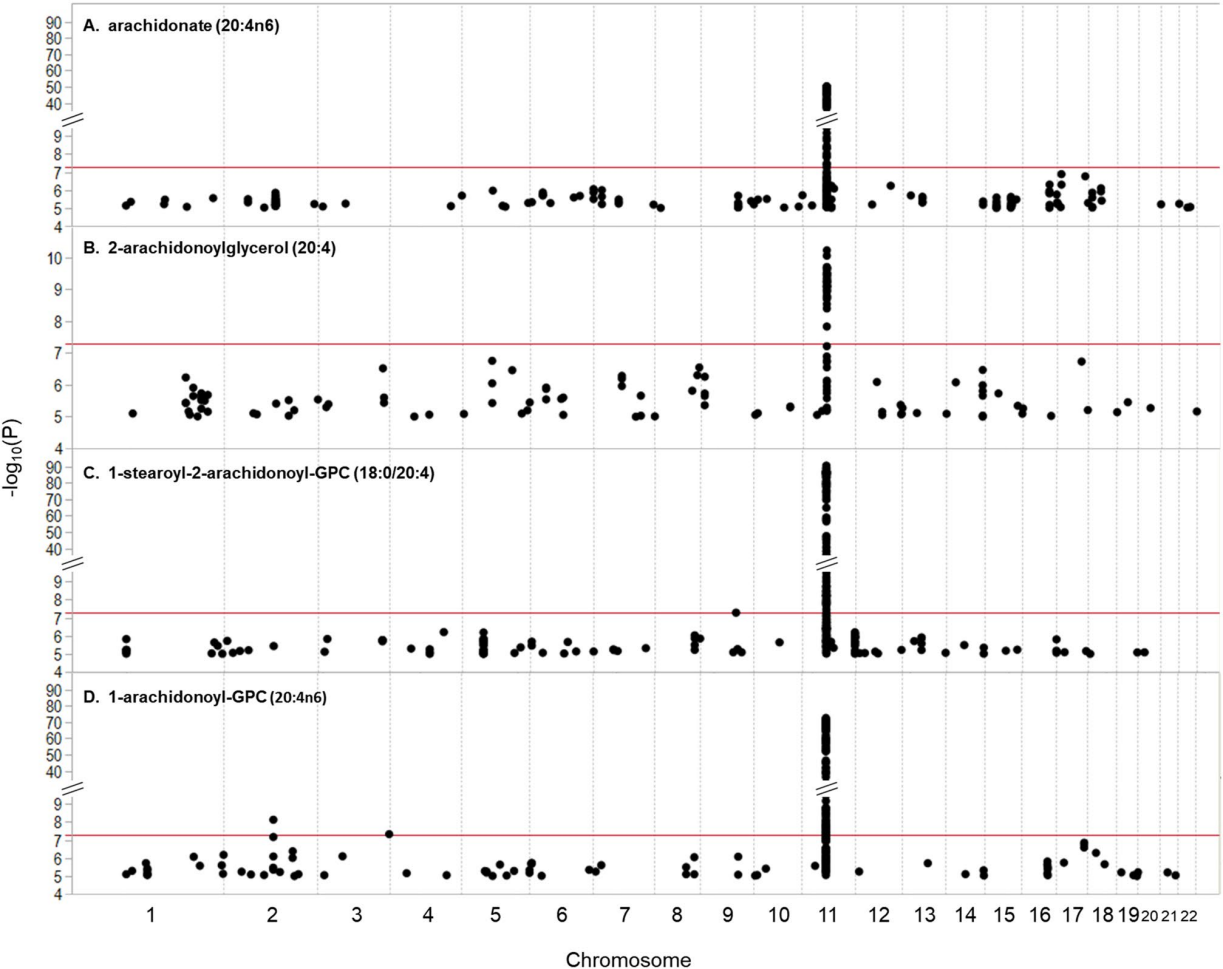
Manhattan plots showing the association of *FADS* locus variation with levels of four n-6 LC-PUFA-containing molecules: ARA, 2-ARA glycerol, 1-stearoyl-2-ARA-GPC and 1-ARA-2-lyso-GPC. The horizontal axis shows the physical location in the genome, organized by chromosome. The vertical axis shows the − log_10_-transformed *p* values from the association analysis with the red line indicating the genome-wide significance level (*p* = 5 × 10^–8^). The four peaks show the strong association with the *FADS* locus.

We investigated the role of genetic variation outside of the *FADS* locus on levels of LC-PUFA molecular species by examining the genome-wide association results for four diverse ARA-containing lipids including unesterified ARA, 2-ARA glycerol, 1-stearoyl-2-ARA-GPC and 1-ARA-2-lyso-GPC (Fig. 3). Surprisingly, all ARA-containing lipids only showed associations with SNPs in the *FADS* locus. The remaining 48 rs174564-associated lipid molecular species listed in Tables 1 and 2 were also examined for associations with genome-wide SNPs. Only 9 of the 48 PUFA- and LC-PUFA-containing lipids were significantly associated with genetic variants outside of the *FADS* locus (*p* < 1.2 × 10^–8^, listed in Table 3). SNPs in or near *MBOAT7* were associated with levels of LA- and ARA-containing PI molecular species, which were also reported to be eQTLs for *MBOAT7* and *TMC* by the GTEx project^43^. Other lipids associated with *FADS* variants that also had genome-wide significant associations outside of the locus, and potential functional relationships with gene expression were eQTLs reported for *LIPC*, *SCD, TMEM229B*, which encode for a hepatic lipase, a stearoyl-CoA desaturase enzyme involved in fatty acid biosynthesis, and a transmembrane protein, respectively.

**Table 3:** Nine lipid metabolites are associated with genetic variants on other chromosomes, *p* < 1 × 10^–8^.

Metabolite	Top SNP	Chr	Alt allele; frequency	n	*p* value	Effect size	Nearby gene	eQTL gene*
1-Palmitoyl-2-linoleoyl-GPI	rs8736	19	C; 0.568	1953	8.93 × 10^–20^	0.32	*MBOAT7/TMC4*	*MBOAT7/TMC4*
1-Stearoyl-2-arachidonoyl-GPI	rs641738	19	C; 0.565	1956	2.88 × 10^–18^	− 0.29	*TMC4* (near *MBOAT7*)	*MBOAT7/TMC4*
1-Stearoyl-2-linoleoyl-GPI	rs8736	19	C; 0.567	1955	8.93 × 10^–20^	0.32	*MBOAT7*	*MBOAT7/TMC4*
1-Palmitoyl-2-linoleoyl-GPE	rs1077834	15	C; 0.209	1959	4.61 × 10^–18^	0.41	*LIPC*	*LIPC*
1-Stearoyl-2-linoleoyl-GPE	rs80123226	15	T; 0.299	1959	2.95 × 10^–13^	0.33	Near *LIPC*	-
1-Palmitoleoyl-2-linoleoyl-GPC	rs603424	10	A; 0.179	1959	5.92 × 10^–13^	− 0.35	*PKD2L1/SCD*	*SCD*
1-Arachidonoyl-2-lyso-GPE	rs58310495	12	T; 0.159	1955	3.62 × 10^–11^	0.21	*SLCO1B1*	-
1-(1-Enyl-palmitoyl)-2-arachidonoyl-GPC	rs11158671	14	A; 0.472	1911	3.59 × 10^–10^	− 0.22	*TMEM229B*	*TMEM229B*
1-Arachidonoyl-2-lyso-GPC	rs146373355	2	G; 0.007	1956	7.47 × 10^–09^	− 1.27	Downstream of *HS6ST1*	–

*Any tissue in GTEx (version 7.0).

## Discussion

Results from whole genome sequencing combined with metabolomics facilitated a more complete analysis of the impact of genome-wide genetic variation (including common and low-frequency genetic variants) on the biologically-important structural and signaling lipid molecules. These data support the *FADS* locus, and particularly *FADS1,* as a central genetic control point for determining circulating levels of molecular species representing four distinct classes of physiologically-important lipids (phospholipids, lyso-phospholipids, free fatty acids and an endocannabinoid). Most recent studies have described the biochemistry and genetics of LC-PUFAs biosynthesis in the context of an oversimplified pathway of *FADS1/2* and *ELOVL5/2*-encoded desaturation and elongation steps that produce unesterified LC-PUFAs (Fig. 1; blue arrows). In fact, it is typically LC-PUFA-containing complex lipids, intermediates and signaling metabolites such as PLs, lyso-PLs, endocannabinoids, and eicosanoids that have vital structural and signaling functions within physiological systems. Even when unesterified LC-PUFAs and their metabolites impact cellular function, they are not directly derived from this desaturation-elongation pathway, but several biochemical steps (Fig. 1; green arrows, purple arrows) downstream after they have been incorporated into PLs and then mobilized by phospholipases. Importantly, genetic association studies to date have largely utilized an analysis approach in which total lipid fractions, or in some cases phospholipids, are isolated, and then, all fatty acids including PUFAs and LC-PUFAs are removed from complex lipids (typically a glycerol lipid or cholesterol backbone) by a saponification step^39^. This in turn leaves the PUFA or LC-PUFA for analysis but leaves no evidence as to the biologically-relevant lipid molecular species most impacted by variation in the *FADS* locus. Additionally, since fatty acids were removed from total lipid or phospholipid fractions in those studies, little information is available to determine whether variants in the genes that encode the numerous non-*FADS* encoded biochemical steps (such as those shown in Fig. 1) are important in regulating lipid molecular species levels. Gieger and colleagues^44^ initially addressed this complex issue by examining associations between genetic variation and 363 metabolites in the serum of 284 male participants. Several SNPs in a linkage disequilibrium (LD) block containing the *FADS1* gene were strongly associated with a number of LC-PUFA-containing phospholipid molecular species. The strength of these associations were shown to be dramatically increased when product/precursor flux (ratios) at the FADS1 enzymatic step were examined. This and a subsequent study^45^ demonstrated large effect sizes for the *FADS1* gene variant associations and showed common genetic variation in *FADS1* have major effects in the “metabolic make-up” of individuals in human populations.

We have built upon these studies by utilizing large-scale published data where whole-genome sequencing and more complete metabolomic profiling was conducted in 1,960 adult participants^40^. Metabolomics provided an opportunity for us to examine enzyme substrates and products in such a way that the metabolome serves as a surrogate measure of enzyme activities responsible for lipid molecular species^44^. The most significant associations identified were located within the *FADS* locus. *FADS1* encodes for the ∆-5 desaturase enzymatic step in the LC-PUFA biosynthetic pathway (Fig. 1). Individual minor allele variants were strongly associated with accumulation of LA and DGLA, precursors to the FADS1 and FADS2 enzymatic steps respectively, as found in phospholipid and lyso-phospholipid molecular species. In contrast, *FADS* minor allele variants were associated with lower levels of phospholipids, lyso-phospholipids, free fatty acids and an endocannabinoid containing n-6 LC-PUFAs (ARA and adrenic acid), n-3 LC-PUFAs (EPA and docosapentaenoic acid), and a n-9 LC-PUFA (mead acid). It is not possible to discern the impact of the FADS1 and FADS2 steps individually due to the genetic architecture of the *FADS* locus, which has long regions of LD throughout. However, the biochemical pattern demonstrating elevated levels of precursor PUFAs and LC-PUFAs upstream of the FADS1 (∆5 desaturase) step are associated with *FADS* minor allele variants and lower levels of LC-PUFAs downstream of the FADS1 step suggests that the FADS1 step is the major biosynthetic event impacted by variation in the *FADS* locus. This observation of *FADS* locus variation impacting the FADS1 biochemical step is consistent with other studies^15,44^. Notably, there were a few exceptions to the observed pattern, including 1-arachidonyl-2-lyso-GPE in which an ARA-containing lyso-phospholipid was directly associated with some *FADS* minor alleles (e.g. rs174564, effect size, SE = 0.59, 0.24) and inversely associated with other *FADS* minor alleles (e.g. rs174529 effect size, SE = − 0.89, 0.24). Given the high LD of rs174564 and rs174529 (R^2^ = 0.933 in 1,000 genomes (GBR) British in England and Scotland population), these bidirectional effect directions were unexpected. Additional studies will be necessary to better understand why some of the metabolites, including 1-arachidonyl-2-lyso-GPE, appear to have a bidirectional associations with *FADS* variants in high LD.

Perhaps the most important finding of this study, from a metabolism perspective, is that the *FADS* locus has the strongest genetic impact on the identified LC-PUFA-containing lipids including phospholipids, lyso-phospholipids, free fatty acids, and an endocannabinoid, with minimal to no association outside of this locus. For example, the *FADS* locus is the only region to associate at genome-wide significance with levels of biologically-important and diverse forms of ARA-containing molecular species including unesterified ARA, 2-ARA glycerol, 1-acyl-2-ARA-GPC, and 1-ARA-2-lyso-GPC (Fig. 3). This is unexpected because a molecule such as 2-ARA glycerol is formed from ARA-containing phospholipids (catalyzed by several steps after the FADS1 enzyme step; green arrows) utilizing phospholipase D + phosphatase steps or after PIP kinase + phospholipase C + diglyceride lipase steps (Fig. 1; orange arrows). In other words, the biosynthesis of this endocannabinoid requires 6–8 additional biochemical steps beyond the FADS1 enzymatic step that initially formed the ARA from DGLA. While Fig. 1 outlines the metabolism of only ARA-containing lipids, similar pathways (utilizing many of the same enzymes) could be drawn for the n-3 (eicosapentaenoic and docosapentaenoic acids) and n-9 (mead acid) LC-PUFAs.

Importantly, only 9 of the 48 LC-PUFA metabolites in association with the peak-associated SNP rs174564 have genome-wide significant signals with genetic loci outside of the *FADS* locus. Of note, *MBOAT7,* which encodes for lyso-GPl acyltransferase that has specificity for arachidonoyl-CoA as an acyl donor in the remodeling of phosphatidylinositol (Fig. 1), was associated with linolenoyl- and arachidonoyl-containing GPI molecular species. These data, which utilize genome-wide common and low-frequency genetic variants, provide confidence that the original study upon which our inferences are made is robust. Furthermore, the current study considered a high proportion of the DNA sequence variation in the genes that could impact LC-PUFA lipid levels and validates the central role of *FADS* variation in the formation of biologically-important lipids.

**Figure 4:**
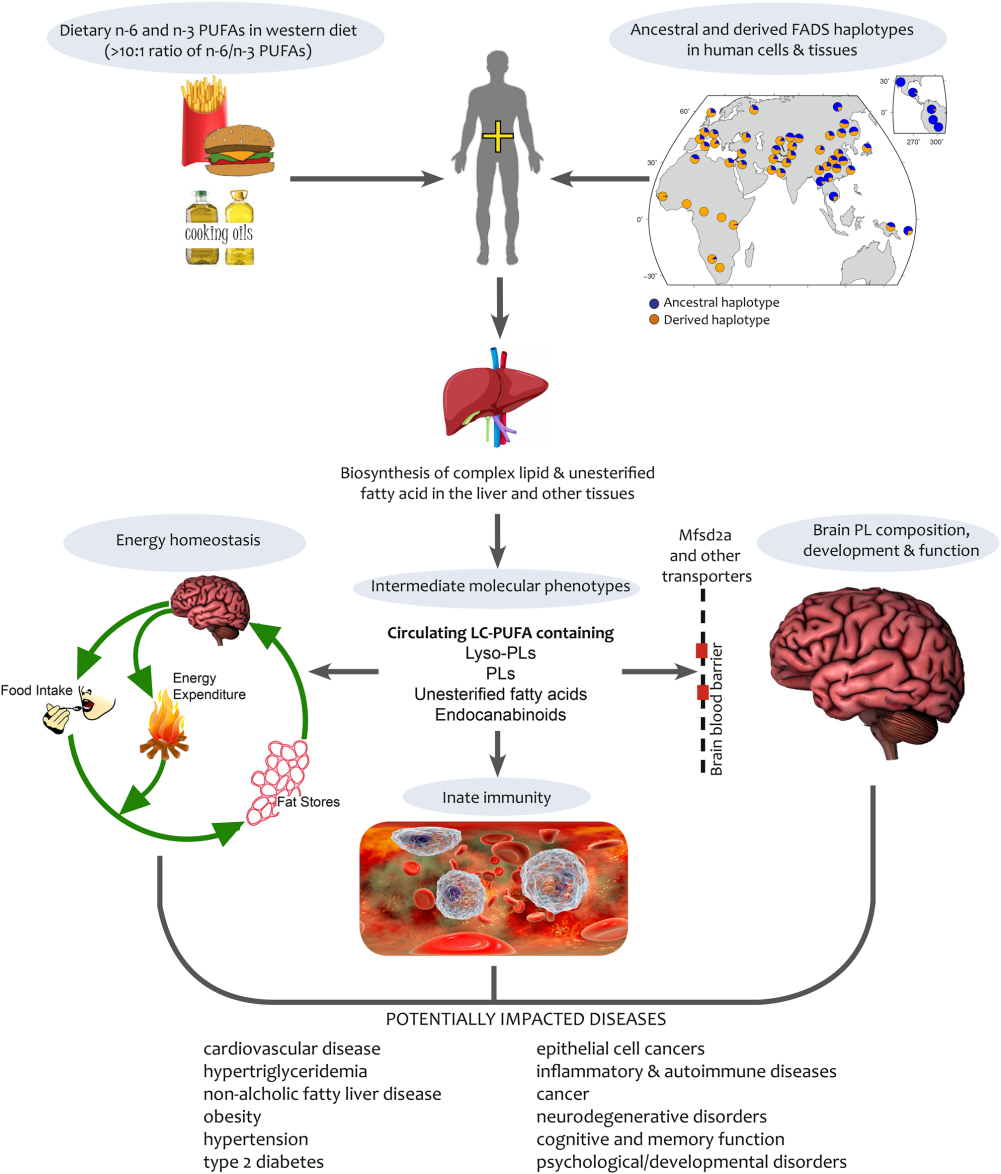
Impact of *FADS* Gene* Dietary PUFA Interaction on energy homeostasis, innate immunity and brain development, memory, and cognitive function. This figure illustrates how levels of PUFAs in western diets^20,57^ interact with global variation within the *FADS* locus to impact levels of LC-PUFA-containing lysophospholipids (lyso-PL), phospholipids (PL), unesterified fatty acids, and endocannabinoids. In turn, circulating and tissue levels of these lipid molecular species impact energy homeostasis^58^, innate immunity^59^, and brain PL composition, development, and function, potentially impacting a wide variety of human diseases^60^.

With regard to the physiological importance of the lipids impacted by *FADS* variation, circulating endocannabinoids and particularly 2-AG plays a critical role in almost all physiological processes, including energy balance and metabolism, thermoregulation, appetite, immune function, stress responses, memory, physical activity, reproduction, and sleep (Fig. 4). The necessity to precisely regulate levels of 2-AG and orchestrate such a wide variety of functions must have been created by intense evolutionary pressures. Fumagalli and colleagues compared signals of selection in an Inuit population and European and Chinese populations to better understand how humans adapted to the Arctic environment^19^. Variants in the *FADS* cluster were the strongest targets of selection and were also strongly associated with multiple metabolic and anthropomorphic phenotypes (smaller body size and shorter stature). These investigators speculated that *FADS* variation led to changes in LC-PUFAs, which in turn affected levels of growth hormones resulting in anthropomorphic changes. While this may be the case, the current study suggests that the direct pathway association of *FADS* variation with 2-AG levels could also be a key molecular mechanism linking the *FADS* locus to several of the aforementioned metabolic and anthropomorphic phenotypes (Fig. 4).

The brain is highly enriched with n-3 and n-6 LC-PUFAs-containing phospholipid pools^46–48^, and studies to date suggest the brain has a limited capacity to synthesize these n-6 and n-3 LC-PUFAs. Consequently, most LC-PUFAs are thought to move from circulation across the blood brain barrier. Recent studies reveal that much of the acquisition of LC-PUFAs by the brain is mediated by a transporter, Mfsd2a, which moves n-3 and n-6 LC-PUFAs as lyso-GPCs from circulation across the blood brain barrier^49–53^. The current study shows that *FADS* variants are strongly associated with levels of n-6 and n-3 LC-PUFA-containing lyso-GPCs, including 1-arachidonoyl-2-lyso-GPC, 1-lyso-2-arachidonoyl-GPC, 1-docosapentaenoyl-2-lyso-GPC, and 1-eicosapentaenoyl-2-lyso-GPC, which are substrates for the Mfsd2a transporter. Taken together, these data suggest *FADS* variation has the capacity to alter the content of brain phospholipids (Fig. 4).

The other two lipid classes impacted by *FADS* variation are n-6 and n-3 LC-PUFAs within phospholipids or as unesterified fatty acids. Levels of LC-PUFA-containing phospholipids within membranes impact a wide range of structural and communication functions. This occurs through numerous mechanisms such as affecting membrane fluidity and serving as substrates for large families of phospholipases (such as PLA_2_, PLA_1_, PLC, and PLD) in order to generate metabolic intermediates for a wide assortment of signaling molecules (Fig. 1; orange, green and purple arrows). The current study confirms that *FADS* variation is associated with both levels of LC-containing PLs and unesterified LC-PUFAs, which is linked to levels of eicosanoids in stimulated human blood^54^.

Dramatic ancestral-based differences in genetic variation within the *FADS* locus have been identified with an ancestral haplotype (minor alleles in this study) associated with reduced capacity to synthesize LC-PUFAs. This haplotype is nearly fixed in Native American and Greenland Inuit populations, elevated in Amerindian-Ancestry Hispanic populations, and virtually absent in African populations^20^. In contrast, there is also a derived haplotype (major alleles in this study) that is associated with higher levels of LC-PUFAs, which is fixed in Africa, elevated in African Americans, and is observed at varying frequencies (25–50%) in Europe and East Asia^18–22,55^. A rapidly emerging literature indicates that the *FADS* locus, and thus the efficiency of LC-PUFA biosynthesis, is an evolutionary ‘hotspot’ that has been targeted numerous times by positive selection during human evolution. The current study illustrates the genetic impact of the *FADS* locus and particularly the FADS1 ∆-5 desaturation step on the formation of molecular species from four biologically-crucial classes of LC-PUFA-containing lipids. These affect a wide diversity of functions including innate immunity, energy balance, and brain development (Fig. 4). This study also illustrates the potential for rapid nutritional transitions, such as modern diets that induce broad evolutionary discordance driven by numerous molecular mechanisms that in turn impact a wide range of diseases/disorders. These transitions may then provide the framework for a better understanding of the molecular underpinnings behind gene-diet interactions that contribute to the biology of racial/ethnic health disparities.

## Materials and methods

Long et al.^40^ looked at associations between 644 metabolites found to be heritable and stable (over three visits) and 6.69 million common (MAF ≥ 0.05) and 4.66 million low-frequency (0.05 > MAF ≥ 0.005) genetic variants in 1960 adults of European descent enrolled in the TwinsUK registry^56^, using a linear mixed model to account for family structure in the cohort and adjusting for sex and age. Metabolites were measured using non-targeted metabolomics. Analysis was performed at Metabolon (Durham, North Carolina, USA) using ultra-high-performance liquid chromatography–tandem mass spectrometry (UPLC–MS/MS) instruments. Genetic data was based on sequencing data with more than 30 × coverage^40^. In the current study, we focused on the reported associations between the lipid subset of the metabolome reported by Long et al. in Supplementary Table 3, including 247 lipids measured in 1911–1959 individuals and genetic variants within a 300 kb region, encompassing the *FADS* locus (hg38, chr11:61,692,980–61,992,051). Numerous lipid classes were present, including unesterified 6–22 carbon fatty acids, amino fatty acids, dicarboxylated fatty acids, branched chained fatty acids, monohydroxylated fatty acids, acylcarnitines, lysophospholipids (lyso-PLs; 1-radyl-2-lyso-GPC, GPE, GPA, GPI; 1-lyso-2-acyl-GPC, GPE, GPI), phospholipids (PLs; 1-radyl-2-acyl-GPC, GPE, GPI), monoacylglycerides, diacylglycerides, sphingolipids, bile acids, steroids, and endocannabinoids. Of these, n-6 and n-3 PUFAs and LC-PUFAs largely reside in molecular species of lyso-PLs, PLs, monoacylglycerides, diacylglycerides, and endocannabinoids. Lipids associated (*p* < 1.2 × 10^–8^) with genetic variants were identified from supplemental results reported by Long et al.^40^. In secondary analyses, for all lipid molecular species associated (*p* < 1.2 × 10^–8^) with genetic variants within the *FADS* locus, we examined genome-wide associations (*p* < 1.2 × 10^–8^) reported by Long et al. with these metabolites to identify other potential genetic influences on levels of individual lipid molecular species.

## Data Availability

Data is publicly available in Supplementary Table 3 at https://www.nature.com/articles/ng.3809#Sec26.
